# The signal pathway for the repressive effect of dipyridamole on myofibroblast transdifferentiation

**DOI:** 10.1111/jcmm.14006

**Published:** 2018-11-19

**Authors:** Qiong Yan, Keisuke Ina, Seiichi Chiba, Huixing Wei, Shuji Tatsukawa, Yoshihisa Fujikura

**Affiliations:** ^1^ Department of Molecular Anatomy Faculty of Medicine Oita University Yufu Oita Japan

## 1 INTRODUCTION

Renal fibrosis is the common final pathway of various chronic kidney diseases (CKDs), irrespective of the initial causes of nephropathy. Decline of renal function, glomerular filtration rate, has been exhibited to be highly associated with the progression of tubulointerstitial fibrosis.^1^ Fibrosis is considered to occur via a variety of events, which increase the level of transforming growth factor‐β1 (TGF‐β1), a profibrotic cytokine, in renal tissue. TGF‐β1 induces the emergence of myofibroblasts, myofibroblast transdifferentiation, followed by the accumulation of extracellular matrices such as type I collagen, ie, fibrosis.^2^ The myofibroblast is characterized by the expression of α‐smooth muscle actin (α‐SMA) in the cytoplasm. Thus, α‐SMA is generally accepted as a marker of fibrosis.^3^, ^4^, ^5^ Dipyridamole is in clinical use as an anti‐platelet agent. The reno‐protective effects exerted by dipyridamole have been demonstrated in animal models^6^ and humans with the early stage CKD.^7^ In almost all of these experiments and studies, the amelioration effects of dipyridamole on proteinuria were indicated to be mediated by the repression of platelet aggregation which was generally recognized in CKD.

Dipyridamole increases cAMP levels by inhibiting phosphodiesterase in a variety of cells including platelets. The cAMP acts through three pathways of its downstream: protein kinase A (PKA), exchange factor directly activated by cAMP (Epac),^8^ and cyclic nucleotide‐gated (CNG) ion channels.^9^ In the Epac pathway, the increased intracellular cAMP binds to Epac, a guanine nucleotide exchange factor, followed by activation of the low molecular weight G‐protein Rap. Epac contributes to numerous pathophysiological processes, including proliferation, differentiation, cell adhesion, cell junction formation, and exocytosis. In the present study, the effects of dipyridamole against renal fibrosis, the late stage CKD, were investigated in a system without platelet aggregation. In addition, the mechanism by which dipyridamole exerted its effects was examined.

## 2 MATERIALS AND METHODS

### 2.1 Reagents and cells

H89, a selective PKA inhibitor, was purchased from Sigma‐Aldrich (St Louis, MO, USA). 4‐methylphenyl‐2,4,6‐trimethylphenylsulfone (ESI‐05), a selective Epac inhibitor, N^6^‐phenyladenosine‐3′,5′‐cyclic monophosphate (Phe‐cAMP), a PKA activator, and 8‐bromo‐2′‐O‐methyladenosine‐3′,5′‐cyclic monophosphate‐acetoxymethyl ester (O‐Me‐cAMP), an Epac activator, were obtained from Biolog Life Science (Bremen, Germany). NRK49F cells (normal rat kidney fibroblasts) were provided by the RIKEN BRC through the National Bio‐Resource Project of MEXT, Japan.

### 2.2 Cell culture

NRK49F cells were cultured in D‐MEM supplemented with 10% FBS (JRH Biosciences, Lenexa, KS, USA), 100 IU/mL penicillin and 100 μg/mL streptomycin in a humidified 5% CO_2_‐95% atmospheric air incubator at 37°C. Throughout this study, cells from passages 3 to 8 were used. Five × 10^4^ cells were seeded in 1.5 mL medium with 10% FBS and cultured for 24 hours. After washing, they were preincubated in a serum‐free medium for 30 minutes. Then they were exposed to stimulants and/or inhibitors in the medium with 0.5% FBS.

### 2.3 Immunofluorescence microscopy

In order to identify the cell type, a monolayer culture of the cells was grown on a two‐well Labtek chamber slide (AS ONE, Osaka, Japan). Cell cultures were divided into three groups: the control, the 2 ng/mL TGF‐β1, and the 2 ng/mL TGF‐β1 + 3 μmol/L dipyridamole group. After treatments, the cells of each group were fixed in 4% paraformaldehyde and subjected to indirect immunofluorescence labelling for α‐SMA. In brief, the cells were permeabilized with 0.5% Triton X‐100 in PBS for 20 minutes, washed with PBS, and incubated with the primary antibody against α‐SMA (Sigma‐Aldrich, St Louis, MO, USA; 1:800 dilution) for 2 hours at room temperature (RT). After washing, they were incubated with FITC‐conjugated goat anti‐mouse IgG secondary antibody (Caltag Laboratories, Burlingame, CA, USA; 1:200 dilution) for 1 hour at RT, followed by DAPI nuclear staining. After staining, the cells were rinsed, observed, and photographed using an Olympus BX 60 microscope equipped with epi‐fluorescence optics. As a negative control, the primary antibody was replaced with mouse nonimmune serum.

### 2.4 Western blots

The cells were exposed to inhibitors of cAMP pathways, 5 μmol/L H89 or 3 μmol/L ESI‐05, and activators of cAMP pathways, 30 μmol/L Phe‐cAMP or 10 μmol/L O‐Me‐cAMP for 1 hour before the addition of 2 ng/ml TGF‐β1. They were exposed to 3 μmol/L dipyridamole for 30 minutes before a treatment of TGF‐β1. They were cultured for 48 hours after TGF‐β1 was added. Then cell lysates were prepared by adding ice cold protein extraction reagent (modified RIPA) with proteinase inhibitors. They (10 μg protein) were applied by 10% SDS‐PAGE electrophoresis and blotted to a polyvinylidene difluoride filter membrane. The membranes were incubated with anti‐α‐SMA, or anti‐GAPDH antibody. The membranes were then incubated with appropriate horseradish peroxidase‐conjugated secondary antibodies. Immunoreactive bands were visualized by using the ECL system, and the intensity of the bands was measured by Image Quant TL (GE Healthcare, Buckinghamshire, UK).

### 2.5 Quantitative real‐time PCR

The cell lysates were subjected to RT‐PCR for α‐SMA at 24 hour after the addition of TGF‐β1. Total RNA was isolated from the cells using the RNeasy Mini kit (Qiagen, Hilden, Germany) according to the manufacturer's instructions. cDNA was prepared using the Transcriptor First Strand cDNA Synthesis kit (Roche Diagnostics, Basel, Switzerland). Quantitative RT‐PCR was performed using the UPL Probe PCR Master Mix and analysed with a Light Cycler 96 System (Roche Diagnostics). All gene expression values were normalized using *GAPDH* as a housekeeping gene. The primers of α*‐SMA* used in PCR amplification were from Roche Diagnostics and are as follows: Forward Primer TGCCATGTATGTGGCTATTCA, Reverse Primer ACCAGTTGTACGTCCAGAAGC.

### 2.6 EIA

The amount of intracellular cAMP at 1 hour after addition of dipyridamole was measured by using a cAMP enzyme immunoassay kit from Cayman Chemical according to the manufacturer's instructions.

### 2.7 Statistics

Quantitative data were compared using Wilcoxon's test or Student's *t* test of the software package JMP (SAS Institute, Cary, NC, USA). *P*‐values less than 0.05 were considered statistically significant.

### 2.8 Results and discussion

**Figure 1:**
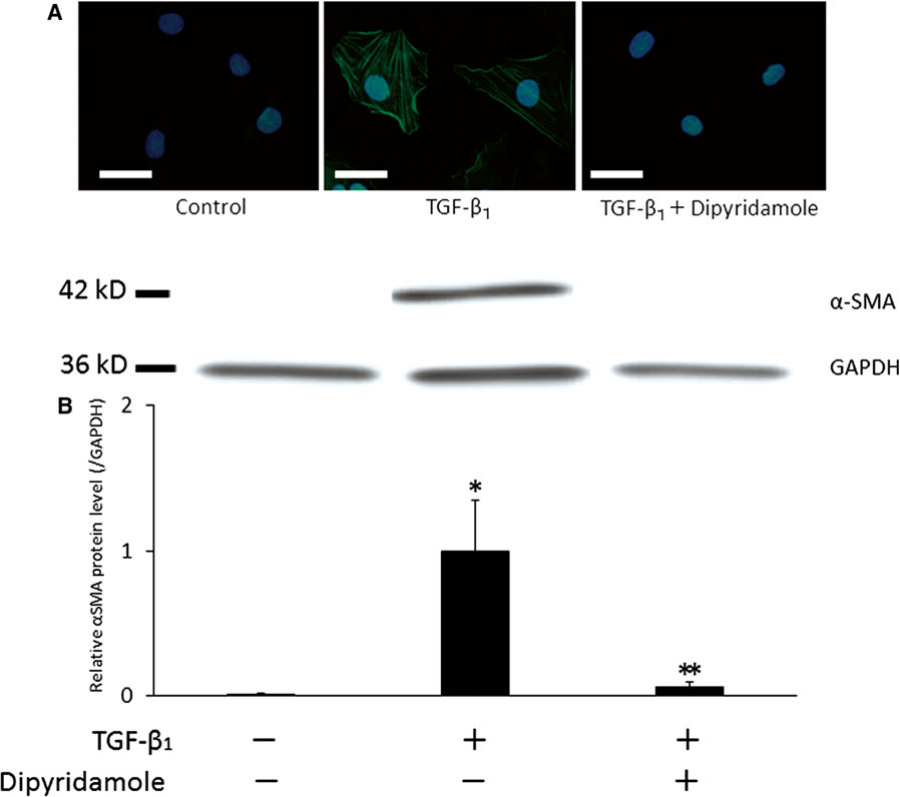
Effect of dipyridamole on expression of α‐SMA. After incubation with the reagents, the cells were subjected to immunofluorescence or Western blots for α‐SMA. A, Immunofluorecence for α‐SMA in the cells treated with or without TGF‐β1 in the presence or absence of dipyridamole. Bar = 30 μm. B, The NRK49F cell lysates from three groups were subjected to immunoblot analysis with antibodies against α‐SMA and GAPDH. The experiments were repeated three times, and one representative blot is shown. GAPDH reprobe is shown to demonstrate lane load. The molecular weights in kD are on the left. Bar graph shows relative α‐ SMA band densities normalized to GAPDH signal.*P < 0.01 vs control;**P < 0.05 vs TGF‐β1 group

**Figure 2:**
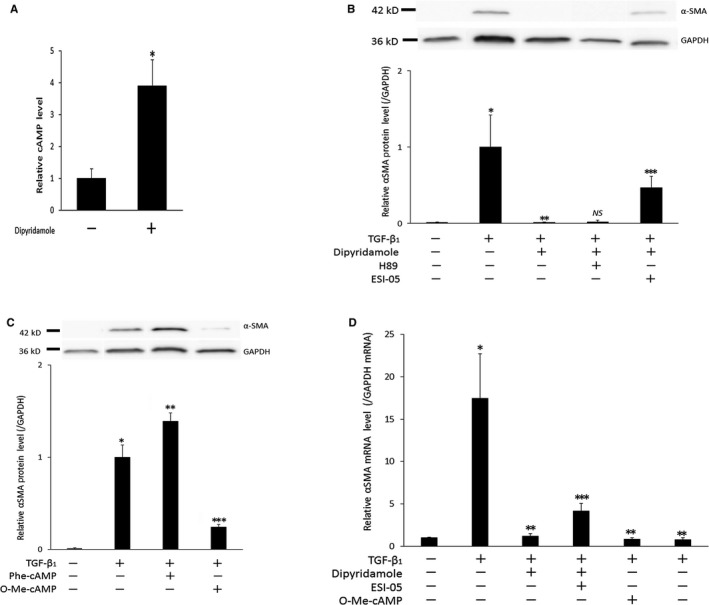
Contribution of cAMP pathways to suppressive effects of dipyridamole on α‐SMA expression. A, Alteration of cAMP levels in the cytoplasm yielded by dipyridamole. After incubation the lysates of the cells were subjected to EIA of cAMP. Bar graph shows relative intracellular cAMP levels. *P < 0.01 vs control. B, Effect of the inhibitor of PKA or Epac pathway on α‐SMA expression. After incubation with the reagents for 48 hours, the cell lysates were subjected to Western blots for α‐SMA. Representative Western blots and relative quantification are provided. *P < 0.005 vs control;**P < 0.005 vs TGF‐β1 group; NS not significant vs TGF‐ β1 + dipyridamole group; ***P < 0.01 vs TGF‐β1 + dipyridamole group. C, Effect of the activator of PKA or Epac pathway on α‐SMA expression. After incubation with the reagents the cell lysates were subjected to Western blots for α‐SMA. Representative Western blots and relative quantification of α‐SMA are shown. *P < 0.001 vs control; **P < 0.05; ***P < 0.001 vs TGF‐β1 group. D, Effect of the inhibitor or the activator of Epac pathway on α‐SMA mRNA expression. The cell lysates were subjected to RT‐PCR. Bar graph shows relative values of α‐SMA mRNA levels normalized to GAPDH signal. *P <* *0.01 vs control; **P < 0.05 vs TGF‐ β1 group; ***P < 0.05 vs TGF‐β1 + dipyridamole group

Immunofluorescence for α‐SMA revealed that TGF‐β1 induced, to a large extent, expression of α‐SMA forming stress fibre (Figure 1A). Dipyridamole repressed TGF‐β1‐induced α‐SMA expression by the normal range (almost none) as shown by immunofluorescence (Figure 1A) and Western blots for α‐SMA (Figure 1B). Dipyridamole suppressed TGF‐β1‐induced myofibroblast transdifferentiation in the in vitro system without platelets (Figure 1). To our knowledge, this is the first report that dipyridamole exerted protective effects against fibrosis corresponding to tubulointerstitial lesions in CKD. Dipyridamole caused the elevation of intracellular cAMP levels (Figure 2A). To determine which pathway of cAMP was involved in the repressive effects of dipyridamole against fibrosis, the cells were exposed to inhibitors or activators of each pathway. H89, a selective PKA inhibitor, did not influence the suppressive action of dipyridamole against TGF‐β1‐induced α‐SMA expression (Figure 2B). Also, N^6^‐Phenyl‐cAMP, a PKA activator, exacerbated TGF‐β1‐stimulated α‐SMA expression (Figure 2C). These results may indicate that even if PKA had been activated through cAMP levels elevated by dipyridamole, it would not have suppressed TGF‐β1‐induced α‐SMA expression. On the other hand, ESI‐05, a selective Epac inhibitor, reduced the suppressive effect of dipyridamole on α‐SMA expression induced by TGF‐β1 (Figure 2B,D). Besides, 8‐Br‐2′‐O‐Me‐cAMP‐AM, an Epac activator, inhibited TGF‐β1‐induced myofibroblast transformation (Figure 2C,D). These data indicated that Epac activation caused by dipyridamole blocked α‐SMA expression stimulated by TGF‐β1. Taken together, the suppressive effects of dipyridamole against increased α‐SMA expression were considered to be exerted via activation of the Epac pathway following elevation of cAMP levels, while the adverse effect of PKA activation was, if present, limited. There are some reports concerning the effects of both pathways in the downstream of cAMP on fibrosis. Generally, both the PKA and Epac pathways suppressed fibrosis.^10^ However, the PKA activator was shown to attenuate fibrosis yielded by exposure of pulmonary fibroblasts to TGF‐β1, while the Epac activator did not affect it.^11^ In contrast, it was demonstrated that the PKA activator induced fibrosis by mesangial cells.^12^ The different effects between the results of the present study and the findings of these reports might be because of the difference in the cells used. The question of which process of the TGF‐β1 signalling pathways did dipyridamole block via Epac pathway activation will be resolved in the next paper.

**Figure 3:**
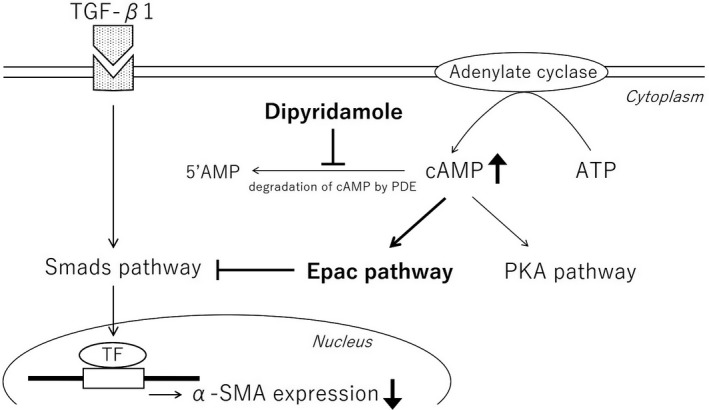
The diagram of the action mechanism of dipyridamole. Dipyridamole inhibits phosphodiesterase, followed by the elevating level of intracellular cAMP. cAMP stimulates Epac pathway, leading to repression of TGF‐β1 signal pathway, Smads pathway, which induces α‐SMA expression. Thus, dipyridamole represses myofibroblast transdifferentiation. PDE, phosphodiesterase; TF, transcription factor

In conclusion, dipyridamole suppressed TGF‐β1‐induced α‐SMA expression via Epac pathway activation. This protective effect of dipyridamole against fibrosis was not mediated by platelet aggregation and was directly exerted on fibroblasts. The Epac pathway provides a new pharmacological approach of dipyridamole for treatment of fibrosis in CKD.

## CONFLICT OF INTEREST

The authors confirm that there are no conflicts of interest.

## AUTHORS CONTRIBUTIONS

QY, KI, and YF conceived and supervised the study; QY, KI, and SC designed experiments; QY, HW, and ST performed experiments; QY, KI, and SC analysed data; QY, KI, and SC wrote the manuscript; QY, KI, and YF made the manuscript revisions.
